# Association of pre-pandemic respiratory system diseases with long COVID: a population-based case-control study

**DOI:** 10.1186/s12879-026-12977-5

**Published:** 2026-03-03

**Authors:** Pia Lindberg, Sebastian Lindblom, Gunnar Ljunggren, Seika Lee, Iryna Kolosenko, Michael Runold, Artur Fedorowski, Caroline Wachtler, Kristina Piontkovskaya, Åsa M. Wheelock, Axel C. Carlsson

**Affiliations:** 1 Division of Immunology and Respiratory Medicine, Department of Medicine Solna and Center for Molecular Medicine L8:02, Karolinska Institutet, Stockholm, 171 76 Sweden; 2 Department of Respiratory Medicine and Allergy, Center for Molecular Medicine, Karolinska University Hospital Solna, Solna, Sweden; 3 Division of Physiotherapy, School of Health, Care and Social Welfare, Mälardalen University, Västerås, Sweden; 4 Division of Family Medicine and Primary Health Care, Department of Neurobiology, Care Sciences and Society, Karolinska Institutet, Stockholm, Sweden; 5 Academic Primary Health Care Centre, Region Stockholm, Stockholm, Sweden; 6 Occupational and Environmental Medicine, Uppsala University, Uppsala, Sweden; 7 Department of Cardiology, Karolinska University Hospital, and Department of Medicine, Karolinska Institutet, Solna, Stockholm Sweden

**Keywords:** COVID-19, Long COVID, Case control studies, Signs and symptoms, respiratory, Somatic symptoms, Symptom cluster, Primary healthcare, Diagnosis

## Abstract

**Objectives:**

Long COVID, defined as diverse symptoms persisting > 3 months post-infection, remains a major post-pandemic healthcare burden. Here we investigate risk factor posed by pre-existing respiratory symptoms and illnesses for development of long COVID, with focus on individuals with mild-to-moderate COVID-19 at the primary infection, that did not require hospitalization during the primary SARS-CoV-2 infection.

**Methods:**

This case-control study was designed to investigate the prevalence of respiratory system-related diagnoses in adult; non-hospitalized long COVID patients (cases) compared to matched controls without a history of long COVID. Data was extracted from the Stockholm Region’s database (VAL) and included diagnoses 12 months pre- and 6 months post-long COVID diagnosis as well as pre-pandemic diagnoses (year 2019). Conditional logistic regression models were applied.

**Results:**

Patients with Long COVID displayed higher frequencies of pre-pandemic respiratory conditions (year 2019) as well as 12 months before long COVID diagnosis compared to controls, including acute upper respiratory tract infections (men: Odds ratio (OR) 2.47, women: OR 2.22), asthma (men: OR 1.76, women: OR 1.95), and bronchitis (men: OR 2.15, women: OR 2.71). ORs for asthma were the highest 12 months before long COVID diagnosis (men: OR 4.18, women: OR 3.76).

**Conclusion:**

Patients with Long COVID with a mild-to-moderate primary SARS-CoV-2 infection had higher prevalence of pre-existing respiratory conditions than controls, suggesting that respiratory diseases including asthma were a significant risk factor for long COVID also in the non-hospitalized population. Understanding the link between common respiratory conditions managed in primary care, including asthma and bronchitis, and long COVID is vital for refining clinical strategies and improving outcomes in post-viral conditions.

**Supplementary Information:**

The online version contains supplementary material available at 10.1186/s12879-026-12977-5.

## Key take-home message

Pre-pandemic respiratory diagnoses, including asthma, were more common among individuals later diagnosed with long COVID compared with matched controls. In line with previous literature, most long COVID cases in our cohort were female.

**Supplementary Information:**

The online version contains supplementary material available at 10.1186/s12879-026-12977-5.

## Introduction

Long COVID also referred to as post-acute Sars-CoV-2 syndrome (PACS) or post-COVID [1, 2] is a new diagnosis with a spectrum of diverse persistent symptoms involving multiple organ systems. Long COVID is defined by symptoms associated with SARS-Cov-2 infection such as chronic cough, chest pain, fatigue, brain fog, dizziness, gastrointestinal symptoms, palpitations, changes in libido and sexual capacity, loss or change in the perception of smell or taste persisting 3 months after the primary infection [3]. In autumn 2020, WHO introduced the first definition and a diagnostic code for post-COVID-19 condition [4]. In 2021, a Delphi process was conducted to provide consensus guidelines for diagnosis of the new post-COVID-19 condition [3, 5]. This consensus specified that symptoms of the syndrome can be diverse and persist for at least three months, can fluctuate in intensity over time and cannot be attributed to any other diagnosis. The risk factors and mechanisms associated with long COVID remain unclear, and it is unlikely that a single factor accounts for the wide range of symptoms affecting different organ systems. At this point it is evident that long COVID is an umbrella diagnosis composed of multiple sub phenotypes, each influenced by distinct risk factors, biological mechanisms, and disease courses. Various elements, such as genetics, age, gender, pre-existing health conditions, microbiome composition, and viral traits, may trigger different pathological responses [6].

Similarities between long COVID and post-intensive care syndrome (PICS) symptoms, and the higher prevalence of long COVID in individuals who received intensive care for severe COVID-19 during the acute infection initially led to the theory that these symptoms primarily represented sequelae of intensive care, common in other conditions [7]. However, a range of studies have indicated a wide variation in prevalence for long COVID. Some studies show long COVID prevalence around 10% among non-hospitalized patients with a mild infection [2, 8]. Longitudinal studies suggest that up to 46% of patients have persistent symptoms 12 months after a non-severe COVID-19 infection [9, 10]. A review by Davies, et al. noted that long COVID affects individuals across all age groups and regardless of the severity of the acute phase [1]. However, the highest incidence of long COVID is seen in those between 36 and 50 years of age, with female predominance, with the majority of cases occurring in non-hospitalized patients who had an asymptomatic or mild-to-moderate initial SARS-COV-2 infection (from here referred to as the non-hospitalized long COVID population) [1].

In this study, we aimed to investigate the association between pre-pandemic respiratory illnesses in primary care settings and long COVID prevalence in the non-hospitalized long COVID population, with a focus on sex differences and asthma prevalence.

## Methods

### Study database

The Stockholm County Healthcare Region (Stockholm Region) provides healthcare services under the governance of the Stockholm Region. With approximately 2.5 million residents, the Stockholm Region is the largest in Sweden, covering a variety of urban and rural areas. Healthcare is meticulously documented in the Stockholm Regional Health Care Data Warehouse (VAL), with a documented credibility through contribution to national health registers and benchmarking reports [11]. Since 1997, diagnoses have been systematically coded according to the World Health Organization’s International Classification of Diseases, 10th revision (ICD-10), ensuring standardization and consistency in healthcare data management [4]. The authors had access to diagnoses recorded at all care levels in the Region, including both primary and specialist care. However, as with any registry-based study, some missing data cannot be ruled out. In addition to registry data, relevant literature on post-COVID respiratory conditions was identified using the CORACLE platform [12].

### Study population

**Fig. 1:**
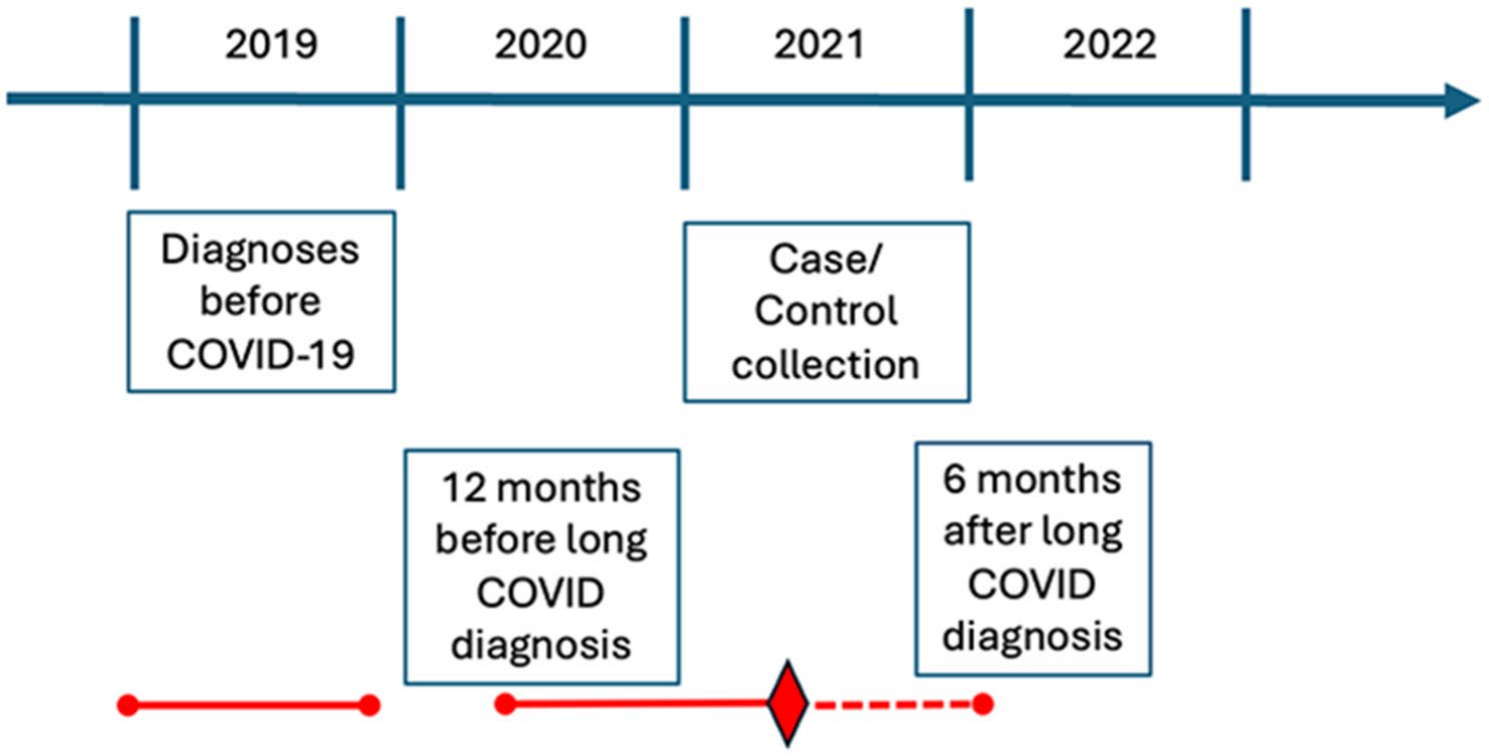
Outline of the study design for the data collection time points. Patients diagnosed with long COVID during 2021 (*n* = 5 589) were matched 1:10 with non-long COVID controls (*n* = 47 561) based on age, sex, and socioeconomical neighborhood on the corresponding date. The timepoint of data collection is outlined for the full study in teal, as well as exemplified for one individual in orange (diamond: date of long COVID diagnosis). Data on respiratory diagnoses were retrieved retrospectively for the Timepoint of long COVID diagnosis, 12 months before long COVID diagnosis, for the pre-pandemic year of 2019, as well as 6 months after long COVID diagnosis

The MIRACLE-S (Multimorbidity Integrated Registry Across Care Levels in Stockholm) study cohort comprised adults 18 years of age and older residing in the Stockholm Region for the entire duration of the study, January 2019 to February 2022 [13]. The source population comprised the general adult population. Long COVID cases were identified based on a registered post-COVID-19 condition diagnosis (ICD-10 U09.9). Controls were sampled from the same underlying population and matched on age, sex, and neighbourhood socioeconomic status, regardless of documented SARS-CoV-2 infection status. Thus, the control group included individuals with undocumented mild-to-moderate SARS-CoV-2 infection as well as individuals without recorded infection, but none had a registered long COVID diagnosis (U09.9). During the first pandemic wave, testing availability was limited and registry-based infection data were incomplete, particularly for mild or asymptomatic infections. Individuals who relocated in or out of the region or died during this period were excluded. The inclusion criteria for long COVID patients (cases) were post-COVID-19 diagnosis U09.9 (ICD-10) obtained for the first time between January 1 and December 31, 2021, in primary or specialized healthcare settings. Individuals who were hospitalized during the acute phase of COVID-19 were excluded. Each case was matched with 10 controls based on age, sex and neighborhood socioeconomic status. Because controls were matched on sex, the study was not designed to estimate sex as an independent risk factor for long COVID within the matched regression models; sex-specific results are therefore presented descriptively and stratified where relevant. Based on the date when cases obtained their long COVID diagnosis, diagnostic codes for symptoms and conditions related to post-COVID with respiratory involvement were extracted [3, 4] from three different timepoints: diagnoses recorded during 2019, 12 months prior to long COVID diagnosis, as well as 6 months after the date of U09.9 diagnosis for both cases and matched controls. Since access to data from the registry was limited until February 10, 2022, the 6-month post-PACS diagnostic timepoint was shorter for some study subjects (minimum of 40 days). The study design is illustrated in Fig. 1.

### Diagnoses

ICD-10 diagnoses applied in this study are presented in Supplementary Table 1 [4]. In brief, the diagnosis code for long COVID is U09.9 (post COVID-19) and the respiratory diagnoses investigated include acute upper respiratory tract infection (J06.9), acute bronchitis (J20.9, J40.9), asthma (J45.1, J45.8, J45.9, J46.9), cough (R05), dyspnea (R06.0) and other and unspecified abnormalities of breathing (R06.8).

### Neighborhood socioeconomic status

The Mosaic tool was utilized to categorize various municipalities and regions within the area based on socioeconomic status. Originally designed to optimize sales efficiency, Mosaic has demonstrated credibility in epidemiological studies due to its ability to generate multivariable models, encompassing over 400 variables. Through the utilization of Mosaic, three levels of socioeconomic status—high, medium, and low—were delineated [14, 15].

### Ethical approval

Ethical approval was obtained from the Swedish Ethical Review Authority (Case no. 2021 − 01016 and 2021–05735).

### Statistical methods

Conditional logistic regression was used to calculate odds ratios (OR) with 99% confidence intervals (CI) for the occurrence of defined diagnoses in cases versus controls. Statistical analysis and data management were conducted using SAS software, version 9.4 (SAS Institute Inc., Cary, NC, USA).

At each time point, the presence of each respiratory diagnosis (yes/no) during the corresponding observation window was compared between cases and matched controls. Conditional logistic regression models were specified consistently across time points, with long COVID case/control status as the dependent variable and each respiratory diagnosis as the independent variable, yielding odds ratios for having the diagnosis among cases relative to controls. The 6-month post-diagnosis window was included to describe respiratory diagnostic patterns after long COVID diagnosis and should not be interpreted as evidence that long COVID causes incident respiratory disease.

For each time window, diagnoses were defined as the presence of at least one registered ICD-10 code during the specified period. The reported frequencies therefore represent the occurrence of recorded diagnoses within each time window, rather than incident disease or point prevalence.

## Results

We identified 5589 individuals (69.1% females), median age 47 years (IQR 39–55) who met the inclusion criteria for cases. These individuals were matched with 47,561 controls. Demographic details are presented in Supplementary Table 2.

**Fig. 2:**
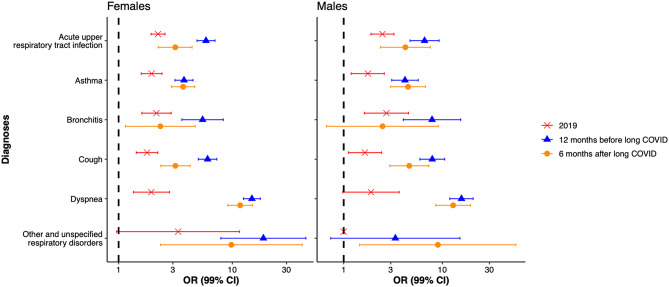
Summary of the association between investigated respiratory symptoms and diagnoses in relation to long COVID. The odds ratios (OR) and 99% CI are presented for females and males respectively in 2019, 12 months prior to long COVID diagnosis, and 6 months after long COVID diagnosis

The odds ratios for the prevalence of the investigated respiratory symptoms and diagnoses between long COVID patients and matched controls for year 2019 (pre-pandemic), 12 months before long COVID diagnosis, and 6 months after long COVID diagnosis are shown in Fig. 2 for both sexes, and in Tables 1 and 2 for females and males respectively.

**Table 1:** Association between long COVID and respiratory system diagnoses in women shown as odds ratios and 99% CI in the year 2019, 12 months before long COVID diagnosis, and 6 months after the long COVID diagnosis. Female Cases: *n* = 3862; Female Controls: *n* = 32,151

Diagnosis	Pre-pandemic (year 2019)	12 months pre-long COVID diagnosis	6 months post-long COVID diagnosis^*^
Other and unspecified abnormalities of breathing R06.8	3.34 (0.96–11.6)	18.8 (7.94–44.4)	9.80 (2.34–41.1)
Acute upper respiratory tract infection J06.9	2.22 (1.93–2.56)	5.87 (4.89–7.05)	3.15 (2.23–4.43)
Bronchitis J20.9, J40.9	2.15 (1.60–2.90)	5.48 (3.60–8.33)	2.33 (1.15–4.72)
Asthma J45.1, J45.8, J45.9, J46.9	1.95 (1.58–2.41)	3.76 (3.13–4.51)	3.69 (2.91–4.68)
Dyspnea R06.0	1.94 (1.35–2.80)	14.9 (12.5–17.6)	11.7 (9.14-15.0)
Cough R05	1.78 (1.43–2.21)	6.06 (5.02–7.31)	3.16 (2.35–4.26)

^*^The 6-month time point was truncated for some individuals due to data access. Please see methods

**Table 2:** Association between long COVID and respiratory system diagnoses in men shown as OR and 99% CI in the year 2019, 12 months before the long COVID diagnosis, and 6 months after the long COVID diagnosis. Male Cases: *n* = 1727; Male Controls: *n* = 15,410

Diagnosis	Pre-pandemic (year 2019)	12 months pre- long COVID diagnosis	6 months post-long COVID diagnosis^*^
Bronchitis J20.9, J40.9	2.71 (1.62–4.54)	7.86 (4.02–15.4)	2.48 (0.67–9.12)
Acute upper respiratory tract infection J06.9	2.47 (1.89–3.23)	6.60 (4.71–9.27)	4.22 (2.36–7.56)
Dyspnea R06.0	1.89 (0.98–3.65)	15.6 (11.9–20.5)	12.9 (8.60–19.3)
Asthma J45.1, J45.8, J45.9, J46.9	1.76 (1.19–2.58)	4.18 (3.07–5.70)	4.49 (2.99–6.73)
Cough R05	1.64 (1.12–2.42)	7.90 (5.92–10.6)	4.62 (2.93–7.31)
Other and unspecified abnormalities of breathing R06.8	0	3.34 (0.74–15.08)	9 (1.45–55.70)

^*^The 6-month time point was truncated for some individuals due to data access. Please see methods

There was a higher prevalence of all studied diagnoses in long COVID patients compared with their matched controls both prior to and after the COVID-19 pandemic (Fig. 2). In the pre-pandemic year (2019), elevated odds ratios were observed for several respiratory diagnoses. Among women, the highest odds ratios were observed for acute upper respiratory tract infection, bronchitis, asthma, and dyspnea, while among men the highest odds ratios were observed for bronchitis and acute upper respiratory tract infection (Fig. 2; Tables 1 and 2). Diagnoses with the highest odds ratio 12 month before long COVID were dyspnea and cough for both sexes, as well as unspecified respiratory disorders for females and bronchitis for males. Notably, the 12-month window prior to the long COVID diagnosis may overlap with the period of acute SARS-CoV-2 infection and/or early post-infectious symptoms, including undiagnosed long COVID. Therefore, this time window should be interpreted as a peri-infection/early post-infection period rather than a purely pre-existing risk window. In contrast, diagnoses recorded during the pre-pandemic year 2019 provide the clearest assessment of pre-infection respiratory morbidity and were therefore emphasized as the primary reference for pre-existing risk factors.

In terms of asthma diagnosis, we obtained OR = 1.95 for females and OR = 1.76 for males in 2019, 3.76 for females and 4.18 for males 12 months before long COVID diagnosis, and 3.69 for females and 4.49 for males 6 months after long COVID diagnosis.

The diagnoses with the highest OR six months after long COVID diagnosis were dyspnea (R06.0) and other and unspecified abnormalities of breathing for both sexes, as well as asthma for females and cough for males (Fig. 2; Tables 1 and 2).

Most of the diagnoses exhibited significantly higher odds ratios, both among females and males 12 months prior to the long COVID diagnosis. Importantly, the odds ratios 6 months after long COVID diagnosis were in general lower compared with the period prior to long COVID diagnosis (Fig. 2; Tables 1 and 2), with the exception of asthma and dyspnea which remained at elevated levels in the long COVID groups of both sexes. This may represent an effect of the correct diagnosis of long COVID having been defined at this time point.

## Discussion

In this study we investigated the frequency of recorded pre-pandemic respiratory diagnoses and related respiratory symptoms as a predisposing risk factor for long COVID. Focus was placed on individuals who did not require hospitalization during their primary SARS-CoV-2 infection who received a long COVID diagnosis in Region Stockholm during 2021, thereby capturing long COVID diagnoses recorded during a period when wild-type and Alpha SARS-CoV-2 variants were predominant at the population level in Sweden. However, because the date of long COVID diagnosis does not correspond to the date of acute infection, individual-level attribution to specific viral variants is not possible and cannot be inferred from these data. Overall, the long COVID group displayed higher odds of recorded respiratory illnesses and symptom diagnoses prior to the primary SARS-CoV-2 infection compared to matched controls. In the pre-pandemic year of 2019, individuals that later developed long COVID had higher odds of acute upper respiratory tract infection, asthma, and bronchitis compared to their matched non-long COVID controls. These associations were observed in both men and women (Fig. 2; Tables 1 and 2). Given potential overlap between the 12-month window and peri-/post-infectious symptom periods, our inference regarding predisposition is primarily based on the pre-pandemic 2019 analyses.

More than two thirds of patients diagnosed with long COVID during 2021 in the Stockholm region were females, thereby corroborating previous findings that long COVID is more prevalent among females [16–20], including non-hospitalized cases. This female predominance is a descriptive characteristic of the cases, as sex was a matching variable in the present case-control design. Furthermore, our study highlights that individuals who developed long COVID following a mild-to-moderate SARS-CoV-2 infection had a higher prevalence of various respiratory symptoms and asthma, identified using ICD-10 codes J45.1 (non-allergic asthma), J45.8 (mixed asthma), J45.9 (unspecified asthma), and J46.9 (status asthmaticus), than their matched controls prior to the COVID-19 pandemic. We found significantly higher odds ratios of asthma among long COVID cases compared to controls, both before and after long COVID diagnosis (Tables 1 and 2). Female sex and asthma have been suggested as risk factors for long COVID in prior studies. In the present study, we observed higher odds of asthma among long COVID cases compared with controls in both women and men across all investigated time windows (Tables 1 and 2). Previous studies have similarly reported associations between female sex, asthma, and the development of long COVID or persistent post-COVID symptoms, supporting the relevance of these factors in non-hospitalized populations [21, 22]. Other work shows similar associations regardless of hospitalization status [19]. Moreover, comorbid conditions such as asthma and a history of healthcare utilization were found to be more strongly associated with long COVID development in non-hospitalized individuals than in those who had been hospitalized for severe COVID-19 [19]. A recently published nationwide observational study utilizing Swedish registries with matched controls also identified the female sex as a risk factor for being diagnosed with long COVID [23]. This study included all individuals with a registered COVID-19 diagnosis, encompassing both hospitalized and non-hospitalized cases [23]. However, limited access to nationwide primary care data may have restricted certain diagnoses. In addition to female sex, the highest risk factors associated with long COVID were dyspnea, abnormal findings on pulmonary diagnostic imaging, and chest pain [23].

Taken together, our data show that patients with long COVID related to mild-to-moderate primary infections have higher proportions of pre-pandemic respiratory symptoms and illnesses than their controls. The elevated incidence of acute respiratory tract infections and bronchitis in the pre-pandemic year among individuals who later developed long COVID should be interpreted with caution. These diagnoses may reflect higher healthcare-seeking behaviour in this population. Alternatively, frequent acute infections may reflect generally increased infection susceptibility and potential immune response deficits, predisposing these individuals to impaired or prolonged reactions to viral infections. In a registry-based setting, such transient diagnoses may act as markers of respiratory symptom burden or healthcare-seeking behaviour, rather than direct causal risk factors for long COVID. Residual predisposition to acute and chronic inflammatory diseases may represent a putative mechanism linking pre-existing respiratory morbidity to the subsequent development of long COVID. Prospective data revealed that baseline respiratory symptoms strongly predicted persistent post-COVID manifestations in outpatient populations, aligning with the pre-pandemic respiratory risk patterns identified in this study [24]. SARS-CoV-2 is a pantropic virus, and pre-infection respiratory conditions may facilitate viral invasion and replication, promote viral persistence and inhibit virus elimination promoting post-viral complications. Further, an impaired immune response to respiratory pathogens that are associated with chronic conditions such as asthma and chronic bronchitis may be also a risk factor for long COVID [25]. Finally, the cumulative effect of prior infections and inflammatory conditions in the respiratory tract might trigger the long COVID cascade, such as is known for asthma. Our observations are in agreement with a study by Al-Aly, et al. demonstrating that individuals with prior respiratory conditions such as asthma and chronic bronchitis are more likely to develop long COVID [6]. This supports the hypothesis that prior respiratory illnesses, such as asthma and chronic bronchitis, may predispose individuals to the development of long COVID, potentially through an increased susceptibility to post-viral complications and chronic inflammation. Liew, et al. recently identified aberrant immune activation, particularly of the IFN-γ/IL-6 pathways, as potential mechanisms linking acute infection to long-term multisystem involvement, suggesting that pre-existing respiratory conditions sharing overlapping inflammatory pathways may predispose to long COVID [26].

Across diagnoses, odds ratios were generally highest in the 12-month period preceding long COVID diagnosis, with diagnosis-specific temporal patterns thereafter. This pattern was most pronounced for bronchitis, while other respiratory diagnoses showed more heterogeneous trajectories over time (Fig. 2). A notable exception was asthma, which remained at approximately doubled odds, while dyspnea remained substantially elevated 6 months after long COVID diagnosis. Thus, long COVID may be associated with an increased likelihood of receiving an asthma diagnosis [6].

However, an alternative explanation is that respiratory symptoms related to long COVID were coded as asthma, reflecting diagnostic misclassification or increased diagnostic intensity rather than true incident asthma. Long COVID is characterized by persistent symptoms such as dyspnea, cough, chest tightness, and variable airflow limitation, which overlap substantially with asthma and may prompt asthma coding in clinical practice, particularly in the context of heightened clinical attention following a long COVID diagnosis [1, 20].

In addition, access to confirmatory lung function testing was limited during the early pandemic due to infection-control concerns related to aerosol-generating procedures. According to international guidelines, including the Global Initiative for Asthma (GINA), spirometry is a key component of asthma diagnosis and management [27]. Reduced availability of spirometry may therefore have increased reliance on symptom-based diagnoses during this period [27, 28]. Consequently, the observed increase in asthma diagnoses following long COVID should be interpreted cautiously and cannot be assumed to represent de novo asthma in all cases.

Notably, despite these diagnostic constraints, we observed elevated odds of asthma diagnoses already in the year preceding long COVID diagnosis (Fig. 2; Tables 1 and 2), suggesting that the association is unlikely to be explained solely by increased diagnostic intensity after long COVID onset. This is consistent with previous observations that baseline respiratory symptoms are predictive of persistent post-COVID manifestations in outpatient populations [24], although diagnostic misclassification cannot be fully excluded.

Viral respiratory infections have previously been associated with subsequent asthma diagnoses in susceptible individuals, particularly among women [6]. In the present study, we observed a similar relative increase in asthma diagnoses in both women and men; however, the mechanisms linking viral infection, persistent respiratory symptoms, and asthma diagnoses remain incompletely understood.

Previous experiences from the H1N1 influenza outbreak, indicated that patients with asthma were at a higher risk for severe illness, including hospitalization. However, early findings suggested that a diagnosis of asthma alone did not increase the risk of SARS-CoV-2 infection or the severity of COVID-19 outcomes. There is growing evidence that different asthma phenotypes play a significant role in assessing the risk of SARS-CoV-2 infection and severity of illness. Studies suggest that type 2 (T2)–high inflammation may lower the likelihood of infection and severe outcomes, whereas type 2 (T2)–low asthma may be associated with an elevated risk [22–24]. Our findings that ICD-10 codes corresponding primarily to non-allergic asthma (J45.1), but also mixed and unspecified asthma (J45.8–J45.9) and status asthmaticus (J46.9), were associated with long COVID support these findings also for the SARS-CoV-2 virus. Our study further shows that asthma is associated with higher risk of persistent symptoms leading to long COVID. This suggests that while asthma may not be a significant driver of acute COVID-19 severity, it poses an increased risk for the development of long-term complications related to long COVID.

Our findings suggest that both female sex and asthma are associated with long COVID, in line with previous literature. Female sex is a well-established risk factor for asthma, which raises the question of how these factors may be related in the context of long COVID. In the present matched case–control design, sex was used as a matching variable, and sex-specific results are therefore presented descriptively. Stratified analyses showed that higher odds of asthma among long COVID cases were observed in both women and men across the investigated time windows (Tables 1 and 2). However, the study was not designed to formally assess independent, additive, or interaction effects between sex and asthma, and such interpretations cannot be made based on these data.

The large population-based cohort and comprehensive healthcare data presented here offer significant strengths, and the matching of cases and controls based on age, gender and socioeconomical area minimizes potential confounding bias. However, our study has limitations that warrant consideration. We used a retrospective study design, yet all diagnoses were registered prospectively in the register we used, limiting information bias regarding the ascertainment of diagnoses. In addition, long COVID was a newly established diagnosis at the time, leading to an underdiagnosis due to a lag in clinical practice, an effect that may have been further accentuated by the general constraints on the healthcare apparatus during the on-going pandemic. As such, it cannot be excluded that many subjects from the control group may have had undiagnosed long COVID. This would bias results toward the null, rather than an overestimate. A further limitation relates to the definition of the source population and control group. Controls were sampled from the general population and were not restricted to individuals with confirmed SARS-CoV-2 infection. During the early pandemic period, access to testing was limited and infection status was incompletely captured in registries, particularly among non-hospitalized individuals. Restricting controls to those with documented infection would therefore have introduced selection bias toward individuals with higher healthcare utilization or more severe acute disease and reduced the population-based representativeness of the study. Importantly, exposure assessment was anchored to the pre-pandemic year of 2019, preceding SARS-CoV-2 circulation, thereby minimizing confounding related to infection risk, testing behaviour, or diagnostic practices. Consequently, our findings should be interpreted as identifying pre-existing respiratory risk factors for being diagnosed with long COVID at the population level, rather than estimating risk conditional on confirmed infection.

An important aspect to consider regarding asthma diagnosis is the impact the pandemic had on the availability of care of this patient group. Access to dynamic spirometry which is essential both for diagnosis and long-term asthma management was limited [29]. The COVID-19 pandemic has had a negative impact on asthma care, with about 25% fewer registrations of asthma and COPD patients in the Swedish Respiratory Registry in 2020 compared to 2019. This suggests that many patients did not undergo the necessary spirometry tests, likely due to the reallocation of healthcare resources and the shift to digital follow-up visits. The risks to healthcare workers related to aerosol formation during spirometry may also have limited the number of spirometry tests performed, particularly for more severe patients. According to the Respiratory Registry, individuals with asthma and COPD did not receive the expected level of evaluations and follow-ups, resulting in fewer asthma diagnoses than usual. The reduced use of spirometry may also be attributed to COVID-19-related guidelines advising against such tests [28]. The global pandemic caused major disruptions in healthcare systems, altering guidelines, medical procedures, and patient interactions. These changes likely contributed to delays in medical assessments and diagnoses, leading to underdiagnosis. The reduced availability of in-person visits, particularly in primary care, may have delayed new diagnoses, and this trend could have extended to other symptoms recorded later as healthcare providers adapted to new protocols. It is also important to recognize that this analysis is based on electronic health records, which often focus on specific reasons for visits and may not fully reflect a patient’s overall health status.

## Conclusions

Our study shows that women and individuals with pre- pandemic respiratory conditions (year 2019), including asthma, had a higher risk of developing long COVID after primary SARS-CoV-2 infection during the first two pandemic waves in the Stockholm Region, representing the wild type and alfa strains. Future research to explore the mechanisms linking pre-existing respiratory diseases to long COVID is needed to identify specific sub-populations of long COVID for better clinical management and targeted interventions.

## Supplementary Information

Below is the link to the electronic supplementary material.

Supplementary Material 1

## Data Availability

The data used in the present study are available for research purposes after ethical approval from Stockholm Region at halsodata. [rst@regionstockholm.se] (mailto: rst@regionstockholm.se).

## Abbreviations

CI: Confidence Interval
COPD: Chronic Obstructive Pulmonary Disease
CORACLE: COVID-19 Research Literature Analysis and Curation Engine
COVID-19: Coronavirus Disease 2019
ICD-10: International Classification of Diseases, 10th Revision
MIRACLE-S: Multimorbidity Integrated Registry Across Care Levels in Stockholm
OR: Odds Ratio
PACS: Post-Acute COVID-19 Syndrome
PICS: Post-Intensive Care Syndrome
SARS-CoV-2: Severe Acute Respiratory Syndrome Coronavirus 2
SES: Socioeconomic Status
VAL: Stockholm Regional Health Care Data Warehouse
WHO: World Health Organization
